# Prevalence and Contributing Factors of Childhood Trauma, Anxiety, and Depression Among Adolescents From Two-Child Families in China

**DOI:** 10.3389/fpsyt.2022.782087

**Published:** 2022-03-18

**Authors:** Jie Tong, Tingting Zhang, Fazhan Chen, Qiang Wang, Xudong Zhao, Manji Hu

**Affiliations:** ^1^Shanghai Pudong New Area Mental Health Center, Tongji University School of Medicine, Shanghai, China; ^2^School of Medicine, Tongji University, Shanghai, China

**Keywords:** childhood trauma, anxiety, depression, adolescents, two-child family, China

## Abstract

The two-child policy has been implemented in China since 2016 and has attracted the attention of the world. Adolescents may face huge psychological challenges in the process of changing family structures and relationships. To date, no mental health survey of adolescents from two-child families has been conducted. We investigated the prevalence and risk factors for childhood trauma, anxiety, and depression in two-child families in a statistically representative sample of Chinese senior high school students from Shanghai. A total of 426 participants were randomly selected from 1,059 students of four senior high schools in different districts of Shanghai. The childhood trauma questionnaire-short form (CTQ-SF), self-rating anxiety scale (SAS) and self-rating depression scale (SDS) were used as the screening tools. We found that the overall prevalence of childhood trauma, anxiety, and depression among senior high school students in two-child families was 46.70% (95% CI, 39.93–53.47%), 22.17% (95% CI, 16.53–27.81%), and 35.85% (95% CI, 29.34–42.36%), respectively. The two-child families was an important factor associated childhood trauma, emotional neglect, and physical neglect (χ^2^ = 5.984, *p* = 0.014; χ^2^ = 4.071, *p* = 0.044; χ^2^ = 4.202, *p* = 0.040). Ranking in two-child families was a risk factor for childhood trauma (β = −0.135, *p* = 0.048). Parental preference was a significantly correlated with physical abuse, physical neglect, anxiety, and depression (β = −1.581 to 0.088, *p* < 0.05). Meanwhile, emotional abuse, physical abuse, emotional neglect, and physical neglect of participants in the two groups were positively correlated with anxiety and depression (*r* = 0.195–0.478, *p* < 0.05). There was a significant relationship between sexual abuse and anxiety symptoms in the one-child family group (*r* = 0.161, *p* < 0.05). The findings suggest that the overall prevalence of childhood trauma, anxiety and depression among adolescents from two-child families in China was high. The two-child families and family ranking are important factors associated childhood trauma, while parental preference is related to anxiety and depression. These results highlight an urgent need to be addressed by adolescents' mental health service providers and policy-makers.

## Introduction

Adolescence is a period of behavioral, cognitive, emotional and physical development and thus is potentially a period of vulnerability (1). Currently, the prevalence of mental disorders among adolescents is increasing, and more attention is paid to it (2). According to the World Health Organization (WHO) in 2020, the global population aged 0–14 reached 1.976 billion, accounting for 25.49% of the total population (3). However, accidental death caused by self-harm and suicide represent the third leading cause of death for all adolescents, with approximately 67,000 adolescents dying every year (4, 5). China has 247 million people aged 0–14, accounting for 12.57% of the global population aged 0–14 and ranking second in the world (6). The overall pooled prevalence of total mental disorders among children and adolescents aged 6–18 was estimated at 15.6% in China (7). In contemporary China, the rapid development of the social economy, heavy academic pressure and termination of the one-child policy have changed the traditional family structure and social support system (8). The mental health problems of adolescence, such as academic fatigue, anxiety, compulsion and depression are increasing year by year (9).

In 1982, the state implemented the one-child policy to adapt population growth to economic and social development, that is a couple was encouraged to have one child (10). For 34 years after the implementation of the plan, the Chinese economy developed rapidly and achieved great success, but there were problems such as population aging and low fertility (11). To promote a balanced population development, the state has advocated the two-child plan since 2016; that is a couple is encouraged to have two children (12). At present, second children account for 57% of the total birth population (13). Meanwhile, changes in family structure, intensification of economic burden, ways of living, parents' education and emotional neglect, may cause mental health problems for adolescents from two-child families (14). Gu (15) found that the social anxiety of children in grade 4–6 with siblings was significantly higher than that of only children, and depression was positively correlated with the conflict degree of the sibling relationship. American psychologist Caplan first proposed crisis theory, which considers the birth of siblings to be a pressure event faced by the eldest children in the family (16). Rutter's psychological structure development hypothesis proposes that pressure events, such as family structure transition, are usually considered to lead to developmental changes in psychological functioning, especially the risk of psychopathology (17).

Childhood trauma is an important predictor of mental health problems in adulthood (18). It is related both to a propensity for increased violence later in life and behaviors harmful to health, such as weariness of learning, social disorder, alcohol addiction, physical inactivity, anxiety, depression and self-harm, leading to poor health outcomes, including an increased risk of personality impairment and psychiatric disorders (19). Galletly et al. in a 20-year follow-up study showed that psychotic symptoms were more common in subjects who had experienced childhood trauma; and were associated with higher rates of emotional and behavioral disturbance, childhood adversity, dysfunctional parenting, and alcohol and cannabis abuse (20). Guo et al. (21) found that nearly half of patients with major depressive disorder (MDD) have experienced childhood trauma, which may cause more serious depressive symptoms, a risk of suicide, and cognitive impairment. The response to antidepressants is also worse. With the birth of a second child, an elder child may face childhood trauma such as emotional and physical neglect, or the second child may be excluded by siblings, even facing physical and emotional abuse (22). Apart from the dramatic impact of childhood trauma, anxiety and depression on the person experiencing them, there is also a high social cost to be paid as a result of the individual's poor adjustment and dysfunction in the community. Early support and intervention in children from two-child families may significantly minimize the negative effects of mental health.

At present, there is no research on the mental health of adolescents in two-child families. We do not know the psychological characteristics of this special population and its correlation with the two-child families. By 2030, the birth rate of second children will exceed 60%, and this population will reach 170 million in China (23). There may be more mental health problems related to the factors of the two-child families in the future. This multicenter cross-sectional study investigated the prevalence and contributing factors of childhood trauma, anxiety and depression among adolescents from two-child families in contemporary China.

## Methods

### Study Design

The study was conceptualized as a cross-sectional, multicentre survey. The study was based on the Shanghai Pudong New Area Mental Health Center (PMHC), Tongji University School of Medicine, which has had a dedicated to a fully functioning child psychology lab since 2011. Along with this, we have been running the child psychology clinic every day. The sample size of the study was calculated based on the formula ${\text{N}}_{sample}={\left(\frac{Z_{1-\alpha /2}}{\delta }\right)}^{2}\times p\times \left(1\text{-}p\right)$ (24). The significance level was 0.05, a two-sided test was required, and the expected effective rate was 80%. The sample size of the two-child and one-child families was estimated to be 170 per group. Our study included a sample of 213 participants per group accounting for about 20% losses in follow-up (those dissatisfied with the contents of the investigation or who could not complete the study for other special reasons).

### Participants and Randomization Procedure

Four senior high schools in different districts of Shanghai were randomly selected from October 1 to December 31, 2020. The following inclusion criteria were employed: (1) 15 years old ≦age ≦19 years old; (2) certain visual and auditory resolution without cognitive disorders; (3) capacity to independently complete the self-test scale; (4) both participants and guardians agreed to participate in the survey and signed the informed consent form. The following exclusion criteria were employed: (1) severe visual impairment, limb disability, extracranial trauma or surgical history; (2) developmental delay diagnosis with behavior disorder; (3) participants or guardians who did not sign the informed consent form, or withdrew halfway.

**Figure 1:**
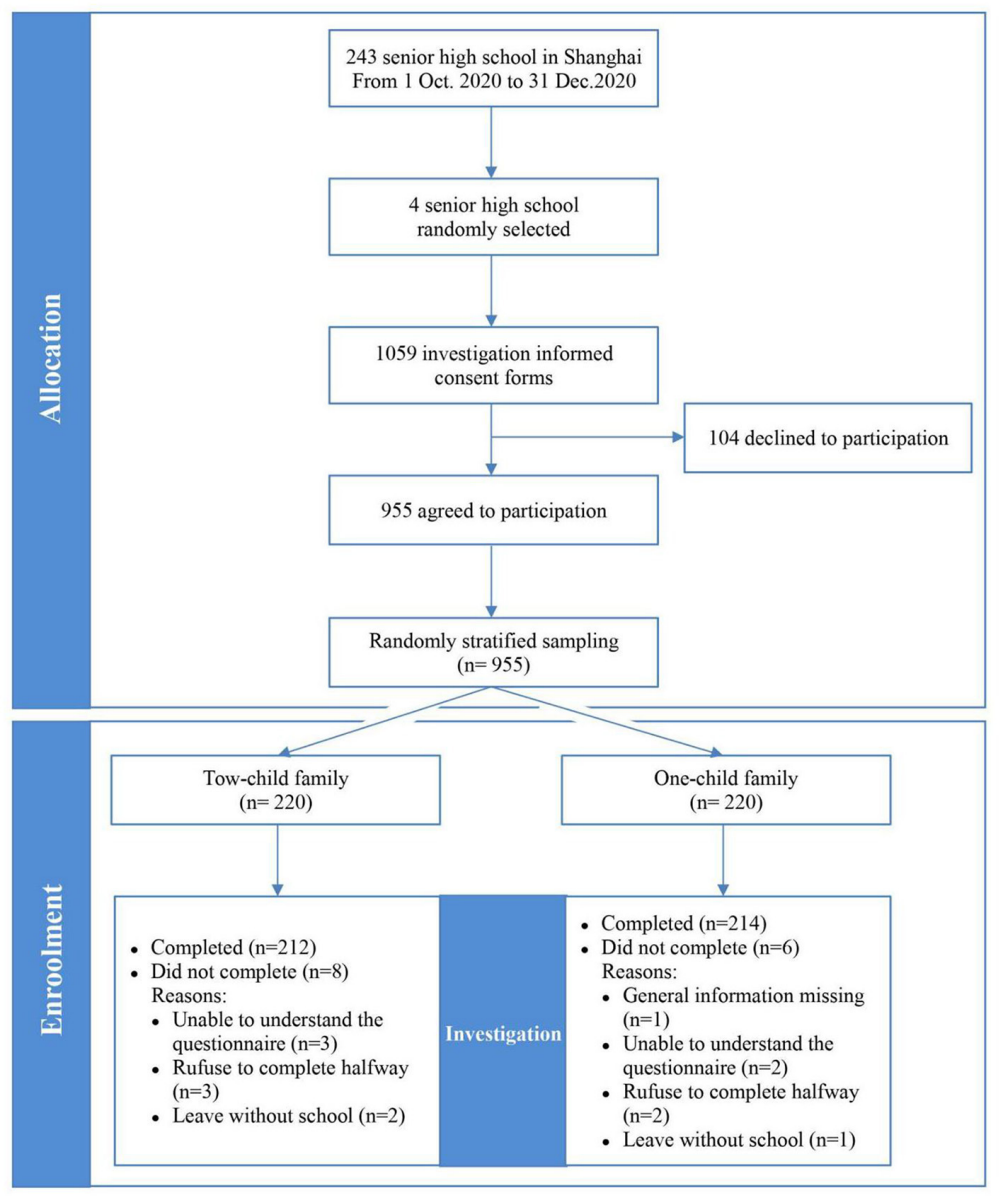
Sample flow diagram depicting the flow of participants through the study.

A total of 1,059 informed consent forms were distributed, and 955 individuals agreed to participate (90.18%). The statistics department of PMHC randomly stratified sampling the selected participants by computer, and 220 participants from the two-child and one-child families were selected. The survey was conducted on students *via* paper questionnaire, and the school psychologist gave unified guidance to the subjects (25). A total of 212 valid questionnaires from the two-child family group and 214 valid questionnaires from the one-child family group were collected. The attrition rates of the two groups were 3.64% (*n* = 8) and 2.73% (*n* = 6) (Figure 1).

### Measures

#### Childhood Trauma Questionnaire-Short Form

The 70-item CTQ scale was proposed by Bernstein et al. in 1994 (26, 27). It is the most commonly used screening measure for trauma and abuse histories of children in both clinical and non-referred groups. Bernstein created a 28-item version of the Childhood Trauma in 2003 (28). Zhao et al. created the Chinese version of the CTQ-SF in 2005, and it provided better reliability and validity. The Cronbach's α coefficient was 0.64 and the remeasuring reliability was 0.75 (29). The CTQ-SF scale included 28 items and was divided into 5 subscales; emotional abuse (EA), physical abuse (PA), sexual abuse (SA), emotional neglect (EN), and physical neglect (PN). The Cronbach's α coefficient of each subscale was 0.55, 0.55, 0.63, 0.65, and 0.16, respectively. Five grades were adopted in each item; 1 point: never true; 2 points: rarely true; 3 points: sometimes true; 4 points: often true; and 5 points: very often true. Of the 28 items, counter-scoring was required on items 2, 5, 7, 13, 19, 26, and 28. Every abuse subscale was scored from 5 to 25 points. When EA ≥ 13, PA ≥ 10, SA ≥ 8, EN ≥ 15, or PN ≥ 10, it is regarded to indicate childhood trauma, as long as one of the above conditions is met (30).

#### Self-Rating Anxiety Scale

The SAS scale was proposed by Zung in 1971 and is used to measure the severity of anxiety (31). Dai et al. created the Chinese version of the SAS in 1986, and it provided better reliability and validity. The Cronbach α coefficient was 0.93 (32). The scale included 20 items, and 4 grades were adopted in each item: 1 point: none or a little of the time; 2 points: some of the time; 3 points: a good part of the time; 4 points: most or all of the time. Of those, counter-scoring was required on items 5, 9, 13, 17, and 19. The standard score was equal to the integer part of the rough score multiplied by 1.25. Participants with mild anxiety had SAS scores of 50–59, those with moderate anxiety had scores of 60–69, and those with severe anxiety had scores of 70 and over.

#### Self-Rating Depression Scale

The SDS scale was proposed by Zung and reflects the subjective feelings of depression intuitively (33). Lee et al. created the Chinese version of the SDS in 1994. The Cronbach's α coefficient was 0.85, and the remeasuring reliability was 0.82 (34). The scale included 20 items, and 4 grades were adopted in each item; 1 point: none or a little of the time; 2 points: some of the time; 3 points: good part of the time; 4 points: most or all of the time. Of those, counter-scoring was required on items 2, 5, 6, 11, 12, 14, 16, 17, 18, and 20. The standard score was equal to the integer part of the rough score multiplied by 1.25. Mild depressions was indicated by SDS score of 53–62, moderate depression by scores of 63-72, and severe depressions by scores of 73 and over.

#### Control Variables

We controlled for sociodemographic factors such as gender (male, female), high school grade (1st, 2nd, 3rd), way of living (alone, with parents, with grandparents, with others), parental marital status (good, ordinary, frequent quarrels, separation, divorce or widowhood), father or mother's education (junior high school and below, senior high school, junior college or above), annual household income (≦$15,000; ≦$15,000 and ≦$45,000; ≦$45,000 and ≦$70,000; ≦$75,000), two-child families or not (yes, no), ranking in two-child families (1st, 2nd), and “Do my parents prefer me in two-child families?” (yes, no).

#### Data Analysis

Data were analyzed using SPSS version 25.0 (SPSS, Inc., Chicago, IL, USA) statistical software. We first identified the prevalence of childhood trauma, anxiety and depression within the sample. Descriptive analysis was performed for sociodemographic data. Data with normal distribution and homogeneous variance were tested by an independent sample *t*-test. Analysis of variance (ANOVA) was used for data that did not obey a normal distribution. The mean and standard deviation were expressed in the form of *x* ± *s*. *Pearson* correlation analysis was used for data subject with a normal distribution, and binary logistic regression analysis was used for categorical variables. The difference was statistically significant at *p* < 0.05.

## Results

### Demographic Characteristics

The demographic characteristics of senior school students from the two-child families, the one-child families, and the total sample in terms of age, sex, grade, way of living, parents' marital status, parents' education, and annual household income are shown in Table 1. There was significant difference in the mother's education between the two groups (χ^2^ = −4.230, *p* = 0.000), but there was no significant difference in other control variables (*p* > 0.05).

**Table 1:** Demographic characteristics of senior school students from two-child and one-child families.

**Variable**	**Total**	**Two-child families**	**One-child families**	***t/x*** **^2^**	***p***
	**(*n* = 426)**	**(*n* = 212)**	**(*n* = 214)**	
Age in years (mean ± SD)	17.00 ± 0.85	16.69 ± 0.74	17.32 ± 0.85	−1.581	0.072
Sex, *n* (%)				−1.952	0.061
Male	181 (42.49%)	75 (35.37%)	106 (49.53%)		
Female	245 (57.51%)	137 (64.63%)	108 (50.47%)		
Grade, *n* (%)				−1.440	0.082
1st	228 (53.52%)	156 (73.58%)	72 (33.64%)		
2nd	111 (26.06%)	38 (17.92%)	73 (34.11%)		
3rd	163 (38.26%)	94 (44.34%)	69 (32.24%)		
Way of living, *n* (%)				−1.240	0.215
Alone	2 (0.47%)	2 (0.94%)	0 (0.00%)		
With parents	357 (83.80%)	171 (80.66%)	186 (86.92%)		
With grandparents	27 (6.34%)	16 (7.55%)	11 (5.14%)		
With others	40 (9.39%)	23 (10.85%)	17 (7.94%)		
Parents' marital status, *n* (%)				−0.790	0.430
Good	307 (72.07%)	149 (70.28%)	158(73.83%)		
Ordinary	62 (56.94%)	33 (15.57%)	29 (13.55%)		
Frequent quarrels	13 (3.05%)	7 (3.30%)	6 (2.80%)		
Separation	2 (0.47%)	1 (0.47%)	1 (0.47%)		
Divorce or widowhood	42 (9.86%)	22 (10.38%)	20 (9.34%)		
Father's education, *n* (%)				−0.273	0.783
Junior high school and below	63 (14.79%)	21 (9.91%)	42 (19.63%)		
Senior high school	120 (28.17%)	77 (36.32%)	43 (20.09%)		
Junior college or above	243 (57.04%)	114 (53.77%)	129 (60.28%)		
Mother's education, *n* (%)				−4.230	0.000^**^
Junior high school and below	61 (14.32%)	26 (12.26%)	35 (16.36%)		
Senior high school	140 (32.86%)	103 (48.58%)	37 (17.29%)		
Junior college or above	225 (52.82%)	83 (39.15%)	142 (66.36%)		
Annual household income, *n* (%)	−1.023	0.306
≦$15,000	90 (21.13%)	41 (19.34%)	49 (22.90%)		
>$15,000 and ≦$45,000	228 (53.52%)	115 (54.25%)	113 (52.80%)		
>$45,000 and ≦$75,000	61 (14.32%)	28 (13.20%)	33 (15.42%)		
>75,000	47 (11.03%)	(13.20%)	19 (8.88%)		

** *p < 0.01*.

### Prevalence

The prevalence of childhood trauma, anxiety and depression among senior high school students from two-child and one-child families in this study is summarized and reported in Table 2. The overall prevalence of childhood trauma among senior high school students was 40.37% (95% CI, 35.70–45.05%), and physical neglect was most common form of maltreatment [25.82% (95% CI, 21.65–29.99%)], followed by emotional neglect [22.77% (95% CI, 18.77–26.77%)]. Physical abuse was the least prevalent at 6.81% (95% CI, 4.41–9.21%). The prevalence of childhood trauma in the two-child family group [46.70% (95% CI, 39.93–53.47%)] was higher than that in the one-child family group [35.05% (95% CI, 28.60–41.49%)], and there was significant difference between them (χ^2^ = 5.984, *p* = 0.014). Among them, physical abuse [7.08% (95% CI, 3.60–10.56%)], emotional neglect [26.89% (95% CI, 20.87–32.90%)] and physical neglect [30.19% (95% CI, 23.96–36.42%)] were higher in the two-child family group than in the one-child family group, while emotional abuse [9.43% (95% CI, 5.47–13.40%)] and sexual abuse [6.13% (95% CI, 2.88–9.39%)] were lower than in the one-child family group. There was significant difference between two groups in the prevalence of emotional neglect (χ^2^ = 4.071, *p* = 0.044) and physical neglect (χ^2^ = 4.202, *p* = 0.040), and there was no significant difference in the rest (*p* > 0.05). Furthermore, the overall prevalence of anxiety and depression among senior high school students was 21.83% (95% CI, 17.89–25.77%) and 33.57% (95% CI, 29.07–38.07%), of which the prevalence of the two-child family group was 22.17% (95% CI, 16.53–27.81%) and 35.85% (95% CI, 29.34–42.36%), higher than that of the one-child family group, but there was no significant difference (*p* > 0.05). There was significant difference in the prevalence of depression severity subgroups between two groups (χ^2^ = 12.008, *p* = 0.002). The heat map of the prevalence of trauma, anxiety and depression among different subgroups of senior high school students was shown in Supplementary Figure 1.

**Table 2:** Prevalence of childhood trauma, anxiety and depression among senior school students from two-child and one-child families.

**Variable**	**Total**	**Two-child families**	**One-child families**	***t/x*** **^2^**	***p***
	**(*n* = 426)**	**(*n* = 212)**	**(*n* = 214)**		
CTQ-SF, mean ± SD	47.19 ± 8.96	46.80 ± 8.21	47.58 ± 9.66	0.900	0.368
Negative/Positive, *n*	254/172	113/99	139/75	5.984	0.014^*^
Prevalence (95% CI), %	40.37 (35.70–45.05)	46.70 (39.93–53.47)	35.05 (28.60–41.49)		
Emotional abuse, mean ± SD	7.58 ± 3.15	7.44 ± 2.86	7.72 ± 3.41	0.920	0.358
Negative/Positive, *n*	382/44	192/20	190/24	0.365	0.546
Prevalence (95% CI), %	10.33 (7.43–13.23)	9.43 (5.47–13.40)	11.21 (6.95–15.48)		
Physical abuse, mean ± SD	5.91 ± 2.17	5.83 ± 1.80	5.99 ± 2.48	0.719	0.472
Negative/Positive, *n*	397/29	197/15	200/14	0.048	0.827
Prevalence (95% CI), %	6.81 (4.41–9.21)	7.08 (3.60–10.56)	6.54 (3.20–9.88)		
Sexual abuse, mean ± SD	5.44 ± 1.93	5.36 ± 1.64	5.51 ± 2.19	0.892	0.407
Negative/Positive, *n*	398/28	199/13	199/15	0.133	0.715
Prevalence (95% CI), %	6.57 (4.21–8.94)	6.13 (2.88–9.39)	7.01 (3.56–10.46)		
Emotional neglect, mean ± SD	10.59 ± 4.47	10.60 ± 4.45	10.57 ± 4.50	−0.251	0.802
Negative/Positive, *n*	329/97	155/57	174/40	4.071	0.044^*^
Prevalence (95% CI), %	22.77 (18.77–26.77)	26.89 (20.87–32.90)	18.69 (13.43–23.96)		
Physical neglect, mean ± SD	7.96 ± 2.72	8.23 ± 2.71	7.70 ± 2.70	−2.038	0.042^*^
Negative/Positive, *n*	316/110	148/64	168/46	4.202	0.040^*^
Prevalence (95% CI), %	25.82 (21.65–29.99)	30.19 (23.96–36.42)	21.50 (15.95–27.04)		
SAS, mean ± SD	42.51 ± 11.13	42.59 ± 11.33	42.44 ± 10.96	−0.134	0.894
Negative/Positive, *n*	333/93	165/47	168/46	0.028	0.866
Mild, *n* (%)	58 (13.62)	28 (13.21)	30 (14.02)	0.876	0.645
Moderate, *n* (%)	24 (5.63)	12 (5.66)	12 (5.61)		
Severe, *n* (%)	11 (2.58)	7 (3.30)	4 (1.87)		
Prevalence (95% CI), %	21.83 (17.89–25.77)	22.17 (16.53–27.81)	21.50 (15.95–27.04)		
SDS, mean ± SD	47.21 ± 13.10	47.47 ± 13.75	46.94 ± 12.43	0.418	0.676
Negative/Positive, *n*	283/143	136/76	147/67	0.985	0.321
Mild, *n* (%)	85 (19.95)	55 (25.94)	30 (14.02)	12.008	0.002^*^
Moderate, *n* (%)	46 (10.80)	18 (8.49)	28 (13.08)		
Severe, *n* (%)	12 (2.82)	3 (1.42)	9 (4.21%)		
Prevalence (95% CI), %	33.57 (29.07–38.07)	35.85 (29.34–42.36)	31.31 (25.04–37.57)		

*CTQ-SF, Childhood Trauma Questionnaire-Short Form; SAS, Self-Rating Anxiety Scale; SDS, Self-Rating Depression Scale*.

* *p < 0.05*.

### Analysis of Contributing Factors

The independent variable were “ranking in two-child families” and “do my parents prefer me in two-child families”; anxiety, depression, childhood trauma and five dimensional factors of two-child families were the dependent variables for binary logistic regression analysis. The “ranking in two-child families” was an important factor associated childhood trauma (β = −0.135, *p* < 0.05). In terms of “do my parents prefer me in two-child families”, there was a significant correlation between this factor with physical abuse, physical neglect, anxiety and depression (β = −1.581 to 0.088, *p* < 0.05) (Table 3).

**Table 3:** Binary logistic regression analysis of different contributing factors on childhood trauma, anxiety and depression among senior high school students from two-child families.

**Variable**	**Ranking in two-child families**					**Do my parents prefer me in two-child families**				
	**β**	**Std. error**	**Wald**	***P***	**OR (95%CI)**	**β**	**Std. error**	**Wald**	***P***	**OR (95%CI)**
CTQ-SF	−0.135	0.069	3.853	0.048^*^	0.874 (0.764–1.000)	−0.082	0.109	0.566	0.452	0.921 (0.745–1.140)
Emotional abuse	0.088	0.089	0.981	0.322	1.092 (0.918–1.299)	0.113	0.165	0.469	0.493	1.120 (0.810–1.548)
Physical abuse	0.010	0.131	0.006	0.938	1.010 (0.782–1.305)	−1.581	0.730	4.687	0.030^*^	0.206 (0.049–0.861)
Sexual abuse	0.193	0.131	2.168	0.141	1.213 (0.938–1.568)	−0.147	0.549	0.072	0.789	0.863 (0.294–2.532)
Emotional neglect	0.047	0.064	0.537	0.463	1.048 (0.924-1.189)	0.122	0.105	1.342	0.247	1.129 (0.919–1.387)
Physical neglect	0.088	0.088	1.013	0.314	1.093 (0.920–1.298)	−0.310	0.160	3.772	0.049^*^	0.734 (0.537–1.003)
SAS	0.011	0.021	0.265	0.607	1.011 (0.970–1.053)	0.088	0.031	8.243	0.004^*^	1.092 (1.028–1.160)
SDS	0.015	0.020	0.529	0.467	1.015 (0.975–1.056)	−0.061	0.031	4.025	0.045^*^	0.941 (0.886–0.999)

*CTQ-SF, Childhood Trauma Questionnaire-Short Form; SAS, Self-Rating Anxiety Scale; SDS, Self-Rating Depression Scale*.

* *p < 0.05*.

### Correlation Analysis

Pearson correlation analysis was conducted on the five dimensions of childhood trauma, anxiety and depression among senior high school students from two-child and one-child families. As Table 4 shows, emotional abuse, physical abuse, emotional neglect and physical neglect were positively correlated with anxiety and depression in the two groups (*r* = 0.195–0.478, *p* < 0.05). Meanwhile, there was a significant relationship between sexual abuse and anxiety symptoms in the one-child family group (*r* = 0.161, *p* < 0.05), but there was no significant correlation with anxiety and depression in the two-child family group (*p* > 0.05).

**Table 4:** Relationship between childhood trauma, anxiety and depression among senior school students from two-child and one-child families.

**Variable**	**Two-child families**		**One-child families**	
	**SAS**	**SDS**	**SAS**	**SDS**
Emotional abuse	0.324 (0.000)^**^	0.424 (0.000)^**^	0.356 (0.000)^**^	0.350 (0.000)^**^
Physical abuse	0.195 (0.004)^**^	0.246 (0.000)^**^	0.231 (0.001)^*^	0.253 (0.000)^**^
Sexual abuse	0.026 (0.708)	0.021 (0.764)	0.161 (0.019)^*^	0.125 (0.068)
Emotional neglect	0.440 (0.000)^**^	0.473 (0.000)^**^	0.258 (0.000)^**^	0.478 (0.000)^**^
Physical neglect	0.270 (0.000)^**^	0.280 (0.000)^**^	0.226 (0.001)^*^	0.396 (0.000)^**^

*SAS, Self-Rating Anxiety Scale; SDS, Self-Rating Depression Scale*.

* *p < 0.05*.

** *p < 0.001*.

## Discussion

We examined the prevalence of childhood trauma, anxiety and depression, as measured by the CTQ-SF, SAS and SDS, among senior high school students from two-child families and examined contributing factors in a multicentre cross-sectional study. Our study is the first to pay attention to the mental health problems of adolescents from two-child families, available till date, which is an original in the research direction. We found that the overall prevalence of childhood trauma, anxiety, and depression among adolescents from two-child families in China was high. Regarding childhood trauma, there was significant difference in the prevalence of emotional and physical neglect between the two-child and one-child families. They are somewhat lower than those described in the European report on preventing child maltreatment (35), which reported emotional abuse in 29%, physical abuse in 22.9%, and sexual abuse in 9.6% of participants; however emotional neglect and physical neglect were higher in our study than in theirs, accounting for 18.4 and 16.3% of participants. According to the National Comorbidity Survey-Adolescent Supplement (NCS-A) survey in the U.S., the prevalence of anxiety among 10,123 adolescents aged 13–18 was the highest. Nearly one in three adolescents (31.9%) met the criteria for anxiety disorder, while affective disorder was found in 14.3%, which is different from our study and may be related to cultural backgrounds and the educational model (36).

On the other hand, the prevalence of childhood trauma in different subgroups (when participants were male, in senior grade one, lived alone, had parents who frequently quarreled, had parents with low education, were the first child of two-child families, and reported parents disliking them) were significantly higher than those in other subgroups. The prevalence of anxiety and depression was higher in the groups of students: who lived alone, whose parents were separated, who were the second child of two-child families, and who reported their parents disliked them. We further confirmed the research of Assari and Tanaka. High parental education and family income had significant protective effects on childhood trauma and family conflict was related to high levels of anxiety (37–39). It also supports the hypothesis that the family is a very important social ecosystem affecting the psychological development of adolescents (40).

Furthermore, the social and psychological development of children and adolescents will also be affected by changes in family structure and the coexistence mode of family members (41). Our study found that the two-child families and the ranking of siblings had a significant correlation with childhood trauma, and parental preference had an impact on adolescents' anxiety and depression. Some studies have shown that the function and structure of the family are negatively correlated with the occurrence of childhood trauma (42). It is also an important risk factor for anxiety and depression in adolescents, and there is an interactive effect (43). Earlier studies of Trias's with 419 twins had given and believed that those twins who experienced parental preference had the least total depressiveness, while twins in the intermediate situation had the highest self-confidence (44). This is consistent with our findings, but there are still few studies on adolescent mental health in multiple birth families.

Emotional abuse, physical abuse, emotional neglect and physical neglect in only children and children with siblings were positively correlated with anxiety and depression, but sexual abuse in the one-child family group was significantly correlated with anxiety symptoms. Humphreys et al. found that childhood trauma was positively associated with depression diagnosis and scores, with emotional abuse and emotional neglect demonstrating the strongest associations in a meta-analysis of 192 unique samples from 190 studies, consisting of 68,830 individuals (45). Importantly, individuals with greater childhood traumatic experiences had a tendency to use more overall maladaptive cognitive emotion regulation strategies, which mediated the relationship between early-life traumatic experience and current anxiety symptoms (46). In addition, one possibility for no significant correlation with sexual abuse, anxiety and depression is that chastity is highly valued in Chinese culture and there is early sex education in the two-child families (47). Our finding is consistent with theoretical and empirical accounts of childhood trauma and depression/anxiety.

## Limitations and Recommendations for Future Studies

We also note several limitations. First, research has been greatly restricted because of the COVID-19 pandemic. The current sample was limited to high school students aged 17–19 years. Second, we did not expand more measurement tools for the prediction of adolescent mental health problems. Future studies should further explore the correlation between the mental health status of adolescents from two-child families and family dynamics, family structure and system family function and expand the age range of the sample to follow-up the outcomes after 5, 10, and 20 years. It could provide more solutions for psychologists or psychotherapists.

## Conclusions

In this study, the overall prevalence of childhood trauma, anxiety, and depression among adolescents from two-child families in China was high. The two-child families and family ranking are important factors associated childhood trauma, while parental preference is related to anxiety and depression. These results highlight an urgent need to be addressed by adolescent' mental health service providers and policy-makers, and provide them with more solutions.

## Data Availability Statement

The original contributions presented in the study are included in the article/Supplementary Material, further inquiries can be directed to the corresponding authors.

## Ethics Statement

The studies involving human participants were reviewed and approved by the Research Ethics Committee of the Shanghai Pudong New Area Mental Health Center and Tongji University Mental Health Center (No: PDJWLL2019008). Written informed consent to participate in this study was provided by the participants' legal guardian/next of kin.

## Author Contributions

JT and TZ: data analysis and writing—original draft preparation and revising. MH and XZ: conceptualization and writing—review and editing. FC: methodology, analysis, and interpretation. QW: supervision. MH: project administration. JT, TZ, and MH: sample collection. All authors approved the submitted version of the manuscript.

## Funding

This work was supported by grants from the Foundation of the Shanghai Municipal Commission of Health and Family Planning (Funding No: 201940161) and the Outstanding Clinical Discipline Project of Shanghai Pudong (Funding No: PWYgy2021-02).

## Conflict of Interest

The authors declare that the research was conducted in the absence of any commercial or financial relationships that could be construed as a potential conflict of interest.

## Publisher's Note

All claims expressed in this article are solely those of the authors and do not necessarily represent those of their affiliated organizations, or those of the publisher, the editors and the reviewers. Any product that may be evaluated in this article, or claim that may be made by its manufacturer, is not guaranteed or endorsed by the publisher.
