# Supplementary material for: Prevalence and Contributing Factors of Childhood Trauma, Anxiety, and Depression Among Adolescents From Two-Child Families in China
Source: Front Psychiatry. 2022 Mar 18;13:782087. doi: 10.3389/fpsyt.2022.782087 (PMC8971896; doi:10.3389/fpsyt.2022.782087)
Supplement: Supplementary file 1 [file Data_Sheet_1.PDF]

Supplement

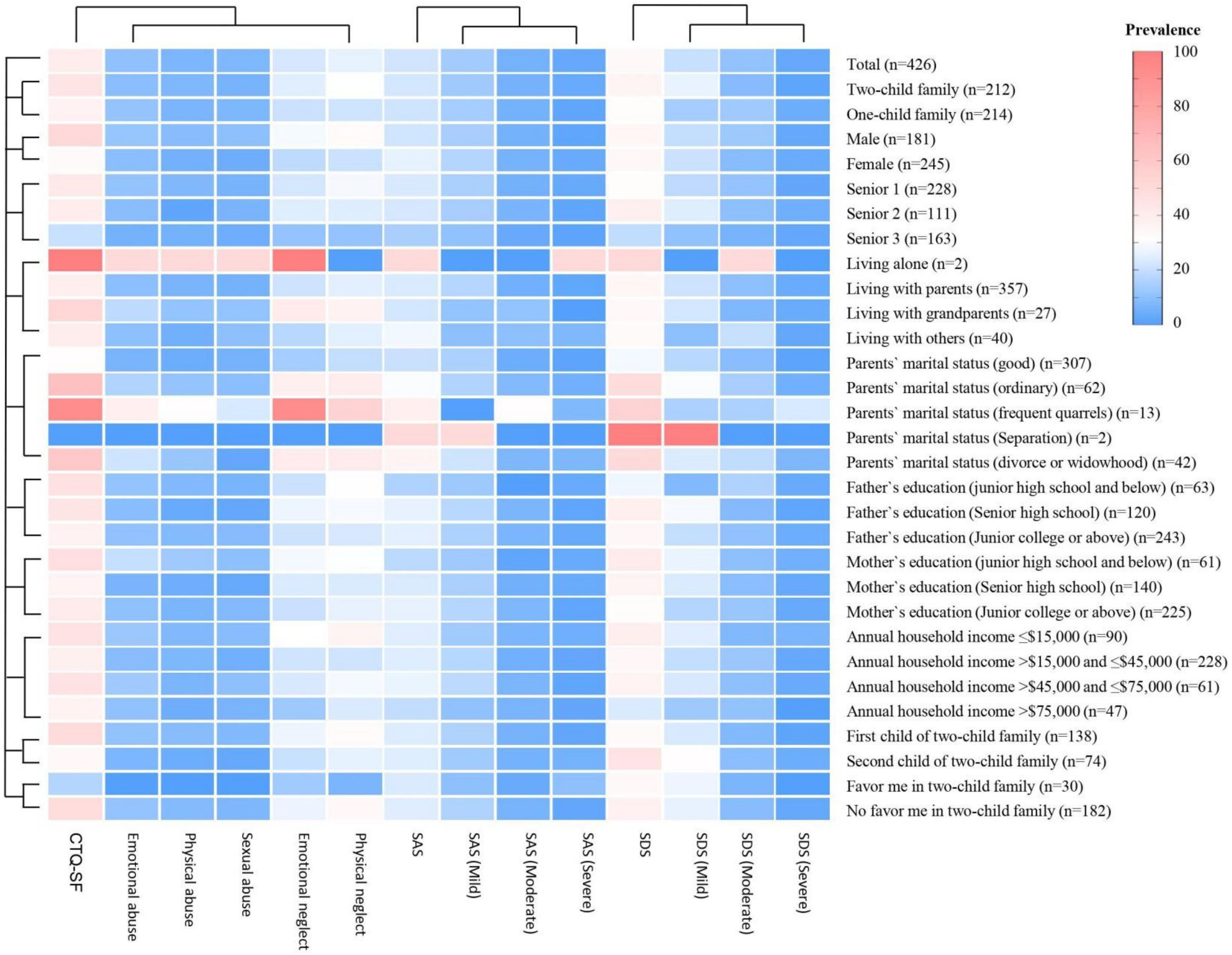

eFigure 1 Heat map of the prevalence of trauma, anxiety and depression among different subgroups of senior high school students.
